# Tuning electronic properties of transition-metal dichalcogenides via defect charge

**DOI:** 10.1038/s41598-018-31941-1

**Published:** 2018-09-11

**Authors:** Martik Aghajanian, Arash A. Mostofi, Johannes Lischner

**Affiliations:** Department of Physics and Materials and the Thomas Young Centre for Theory and Simulation of Materials, Imperial College London, London, SW7 2AZ UK

## Abstract

Defect engineering is a promising route for controlling the electronic properties of monolayer transition-metal dichalcogenide (TMD) materials. Here, we demonstrate that the electronic structure of MoS_2_ depends sensitively on the defect charge, both its sign and magnitude. In particular, we study shallow bound states induced by charged defects using large-scale tight-binding simulations with screened defect potentials and observe qualitative changes in the orbital character of the lowest lying impurity states as function of the impurity charge. To gain further insights, we analyze the competition of impurity states originating from different valleys of the TMD band structure using effective mass theory and find that impurity state binding energies are controlled by the effective mass of the corresponding valley, but with significant deviations from hydrogenic behaviour due to unconventional screening of the defect potential.

## Introduction

Since the discovery of graphene, there has been significant interest in the development of ultrathin devices based on two-dimensional (2D) materials. In contrast to graphene, which is a semimetal when undoped, monolayer transition-metal dichalcogenides (TMDs) with the chemical formula *MX*_2_ (*M* = Mo, W; *X* = S, Se, Te) are semiconductors with a direct band gap^1,2^. Monolayer TMDs have been used as channel materials in field-effect transistors^3,4^ and microprocessors^5^, as well as absorbers in solar cells^6^ and as sensors^7,8^, with promising results.

Defects play a critical role in the performance of devices under realistic conditions^9–11^. Analogously to conventional bulk semiconductors, impurities with shallow donor or acceptor states can be used to control the carrier concentration in TMDs via defect engineering^12,13^. Adsorbed atoms and molecules are a particularly promising class of impurities in TMDs as they tend to only weakly perturb the atomic structure of the TMD substrate, thereby limiting any degradation of carrier mobility that may result from impurity scattering or trapping^14,15^, and experimental fabrication of adsorbate-engineered samples is straightforward^16^.

A detailed theoretical understanding of the properties of charged adsorbates on TMDs is important to enable the rational design of new devices. On the one hand, many groups have used ab initio density-functional theory (DFT) to study the interaction of adsorbed atoms and molecules with TMDs. Such calculations yield important material-specific insights about adsorption geometries, adsorbate binding energies and charge transfer^17–21^. However, ab initio calculations are limited in terms of the size of the systems that can be considered (typically containing up to several hundred or a few thousand atoms), which are much too small to describe properties of shallow defect states that can extend up 100 Ångstrom (Å) or more, as has been observed recently for Coulomb impurities in graphene using scanning tunnelling spectroscopy (STS)^22^.

On the other hand, continuum electronic structure methods, such as Dirac theory for graphene or effective mass theory for bulk semiconductors, can describe the behaviour of extended impurity states, but require parameters from experiments or ab initio calculations, such as Fermi velocities, effective masses^23–27^ and rather importantly, the defect potential that is typically screened by electrons of the host material.

In this paper, we study properties of shallow impurity states induced by charged adatoms on monolayer MoS_2_. Using large-scale tight-binding models and screened defect potentials calculated from ab initio dielectric functions, we reveal a surprising diversity of bound defect states resulting from the unconventional screening present in reduced-dimensional materials and the interplay between multiple valleys in the TMD band structure. We present results for impurity wavefunctions and binding energies as function of the impurity charge and also compute the local density of states (LDOS) in the vicinity of the adatom, which can be measured in STS experiments. For both donor and acceptor impurities, we find that impurity wavefunctions have similar nodal structure to 2D hydrogenic states, but with radii that lie on the nanoscale. We find that that the orbital character of the most strongly bound impurity state switches as a function of the impurity charge strength *Z* due to the different effective masses associated with different valleys in the monolayer TMD band structure. We compare our results to the 2D hydrogen atom and also to effective mass theory calculations and discuss the limitations of these continuum models. Whilst an approach based on the effective mass model is able to describe some of the general behaviour with reasonable accuracy, we find significant discrepancies from our tight-binding model which arise from short-range features of the defect potential. Our calculations demonstrate the potential of adsorbate engineering for ultrathin devices based on TMDs and the importance of first-principles based description of their properties.

## Methods

To describe the electronic structure of the MoS_2_ monolayer, we employ the three-band tight-binding (TB) model by Liu *et al*.^28^. This model uses a basis of transition-metal $4{d}_{{z}^{2}}$, 4*d*_*xy*_ and $4{d}_{{x}^{2}-{y}^{2}}$ orbitals which give the dominant contribution to the states near the conduction and valence band extrema and includes hoppings up to third-nearest neighbours as well as spin-orbit interactions. The various parameters were determined by fits to DFT band structures.

The charged adatom is described as a point charge *Q* = *Ze* (with *e* being the proton charge) located a distance *d* above the plane of the transition-metal atoms. The charge gives rise to a screened potential in the TMD sheet. Within linear response theory, the screened potential is given by

1 $$V(\rho ;\,Z,d)=Z{e}^{2}{\int }_{0}^{\infty }{\rm{d}}q\,{\varepsilon }_{{\rm{2D}}}^{-1}(q){J}_{0}(q\rho ){e}^{-qd},$$

where *ρ* denotes the in-plane distance from the adatom and ${\varepsilon }_{{\rm{2D}}}^{-1}(q)$ is the inverse 2D dielectric function of a single TMD monolayer. The 2D dielectric function can be obtained from the inverse dielectric matrix ${\varepsilon }_{{\bf{GG}}^{\prime} }^{-1}({\bf{q}})$ of an infinite system of stacked TMD sheets (simulated in an electronic structure calculation that employs periodic boundary conditions) via^29^

2 $${\varepsilon }_{{\rm{2D}}}^{-1}({\bf{q}})=\frac{q}{2\pi {e}^{2}{L}_{z}}\sum _{{{\bf{G}}}_{z}{{\bf{G}}}_{z}^{^{\prime} }}\,{\varepsilon }_{{{\bf{G}}}_{z}{{\bf{G}}}_{z}^{^{\prime} }}^{-1}({\bf{q}}){v}_{{\rm{trunc}}}(|{\bf{q}}+{{\bf{G}}}_{z}^{^{\prime} }|).$$

**Figure 1:**
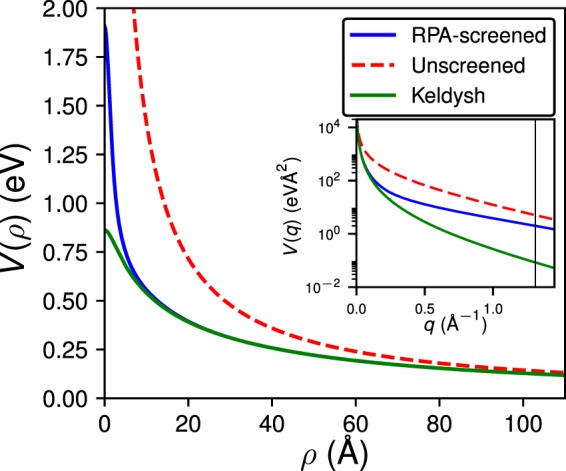
RPA-screened potential of a charged adatom situated *d* = 2 Å above the Mo-atom in MoS_2_ with strength *Z* = 1 (blue solid curve) compared to the unscreened Coulomb potential (red dashed curve). We also compare this to the Keldysh model (green curve) for *Z* = 1, *d* = 2 Å and screening length *ρ*_0_ = 45 Å (Eq. 5), fitted to the RPA-screened potential. The inset shows the Fourier transform of the screened and unscreened potentials, as well as the potential screened in the Keldysh model, with the solid vertical line indicating |**K** − **K**′|, the separation in reciprocal space between the two valleys of MoS_2_.

Here, **G**_*z*_ and ${{\bf{G}}{\boldsymbol{^{\prime} }}}_{z}$ denote reciprocal lattice vectors along the out-of-plane (*z*) direction, *v*_trunc_ is a slab-truncated Coulomb interaction^30^ and *L*_*z*_ denotes the distance between the stacked sheets. The inverse dielectric matrix is computed for a MoS_2_ monolayer using the random-phase approximation^31^ (RPA) with Kohn-Sham wave functions and energies from ab initio DFT (see Supplementary Materials for details). Calculations were carried out using the Quantum Espresso^32^ and BerkeleyGW software packages^33^. For small wave vectors, which are relevant for describing shallow impurity impurity bound states, we find that the right hand side of Eq. (2) depends only on the *magnitude* of the wave vector.Figure 1 shows the screened (calculated from Eq. 1) and unscreened potentials of a charged adatom with *Z* = 1 an*d d* = 2 Å above the Mo-layer in the MoS_2_ sheet. While there are clear differences at short distances, the two potentials both converge to the unscreened case at long distances from the adatom which is characteristic of screening in 2D semiconductors. This short-range discrepancy corresponds to significant differences between the Fourier transforms of these potentials at large wavevectors, shown in the inset of Fig. 1.

To study shallow bound states of the screened adatom potential, we construct a 51 × 51 TMD supercell containing 7803 atoms and diagonalize the resulting TB Hamiltonian with the adatom potential as an on-site term^22,23^. Note that the adatom is placed above a transition-metal site as this is the preferred adsorption geometry for many adatom species, such as alkali metals^17–19^.

To analyze the results of our atomistic tight-binding simulations, we have also carried out calculations using effective mass theory. In this approach, which has been used routinely to study shallow bound states of charged impurities in bulk semiconductors^25,34,35^, the impurity states are expressed as ${{\rm{\Psi }}}_{n\nu }({\bf{r}})=\int {\rm{d}}{\bf{k}}\,{\varphi }_{n\nu }({\bf{k}}){\psi }_{n{\bf{k}}}({\bf{r}})$. Here, *ψ*_*n***k**_ denotes an unperturbed Bloch state with band index *n* and crystal momentum **k** of the host material and *ϕ*_*nv*_(**k**) is an envelope function determined by^36^

3 $${\varepsilon }_{n{\bf{k}}}{\varphi }_{n\nu }({\bf{k}})+\int {\rm{d}}{\bf{k}}^{\prime} \,\langle {\psi }_{n{\bf{k}}}|V|{\psi }_{n{\bf{k}}^{\prime} }\rangle {\varphi }_{n\nu }({\bf{k}}^{\prime} )={E}_{n\nu }{\varphi }_{n\nu }({\bf{k}}),$$

where *ε*_*n***k**_ describes the band structure of the host material and *V*(**r**) denotes the screened impurity potential. In bulk semiconductors, *V* can be accurately approximated^36^ by *Ze*^2^*ε*^−1^(*q* = 0)/*r* and the resulting equation for the impurity state envelope function reduces to the Schrödinger equation of a hydrogen atom with a reduced Bohr radius ${\tilde{a}}_{0}=({m}^{\ast }/{m}_{0})Z{a}_{0}{\varepsilon }^{-1}(q=0)$ (with m* and *m*_0_ denoting the effective and bare mass of the electron, respectively, and *a*_0_ is the Bohr radius). In this approximation, the impurity state envelope functions take the form of the 2D hydrogenic states^37^ give by

4 $${\varphi }_{nl}^{({\rm{2DH}})}(\rho ,\theta )=\frac{{e}^{il\theta }}{{N}_{nl}(Z,{m}^{\ast })}{(\rho {\lambda }_{n})}^{|l|}{e}^{\rho {\lambda }_{n}\mathrm{/2}}{L}_{n-l-1}^{\mathrm{2|}l|}(\rho {\lambda }_{n}),$$

where *N*_*nl*_ is a normalization constant, ${L}_{j}^{k}$ are the generalized Laguerre polynomials, and ${\lambda }_{n}=\frac{2}{2n+1}\frac{Z{m}^{\ast }{e}^{2}}{4\pi {\varepsilon }_{0}{\hslash }^{2}}$. We compare these solutions to the wavefunctions extracted from our TB model to identify similarities in nodal structure.

The screened impurity potential in a 2D semiconductor, such as a TMD monolayer, however, cannot be accurately approximated by a bare Coulomb interaction divided by a *constant* dielectric function (see Fig. 1). A well-known model for the screening of a point charge embedded in a thin dielectric film was derived by Keldysh^38^ and is given by

5 $${\varepsilon }_{{\rm{Keldysh}}}(q)=1+{\rho }_{0}q$$

where *ρ*_0_ is the screening length. We calculate the screened potential *V*_Kelysh_(*ρ*) using the Keldysh model by substituting ${\varepsilon }_{{\rm{Keldysh}}}^{-1}(q)$ for the inverse dielectric function in Eq. 1. The value of *ρ*_0_ = 45 Å is obtained by fitting to the RPA-screened potential of Fig. 1. The Keldysh model has been frequently used to study excitons in TMDs^29,39,40^ and we also use it here for comparison to our tight-binding results.

To simplify the integration over *k*-points in Eq. (3), Bassani *et al*.^36^ divided the first Brillouin zone into subzones Ω_*i*_ centered on critial points **k**_*i*_, typically associated with band extrema. The impurity states Ψ_*nv*_(**r**) are then constructed as linear combinations of subzone states

6 $${{\rm{\Psi }}}_{n\nu i}({\bf{r}})\approx {\varphi }_{n\nu i}({\bf{r}}){\psi }_{n{{\bf{k}}}_{i}}({\bf{r}}).$$

To determine the subzone envelope functions *ϕ*_*nvi*_(**r**), we minimize the expectation value of the Keldysh Hamiltonian $\hat{H}=\frac{-{\hslash }^{2}}{2{m}_{i}^{\ast }}({\partial }_{x}^{2}+{\partial }_{y}^{2})+{V}_{{\rm{Keldysh}}}(r)$ (where ${m}_{i}^{\ast }$ denotes the effective mass associated with the relevant conduction or valence band at **k**_*i*_) using the following ansatz for the most strongly bound impurity state

7 $${\varphi }_{1s,i}(\rho ;\alpha )=\frac{(2\alpha ){e}^{\alpha d}}{\sqrt{2\pi (2\alpha d+1)}}{e}^{-\alpha \sqrt{{\rho }^{2}+{d}^{2}}},$$

where *α* is a variational parameter, which we use to define the impurity radius *a*_imp_ = *α*^−1^. Once the subzone states are obtained, the full impurity states are found by including interactions between different subzones. As the coupling is usually weak, it can be treated using perturbation theory^36^.

## Results and Discussion

### Acceptor States

**Figure 2:**
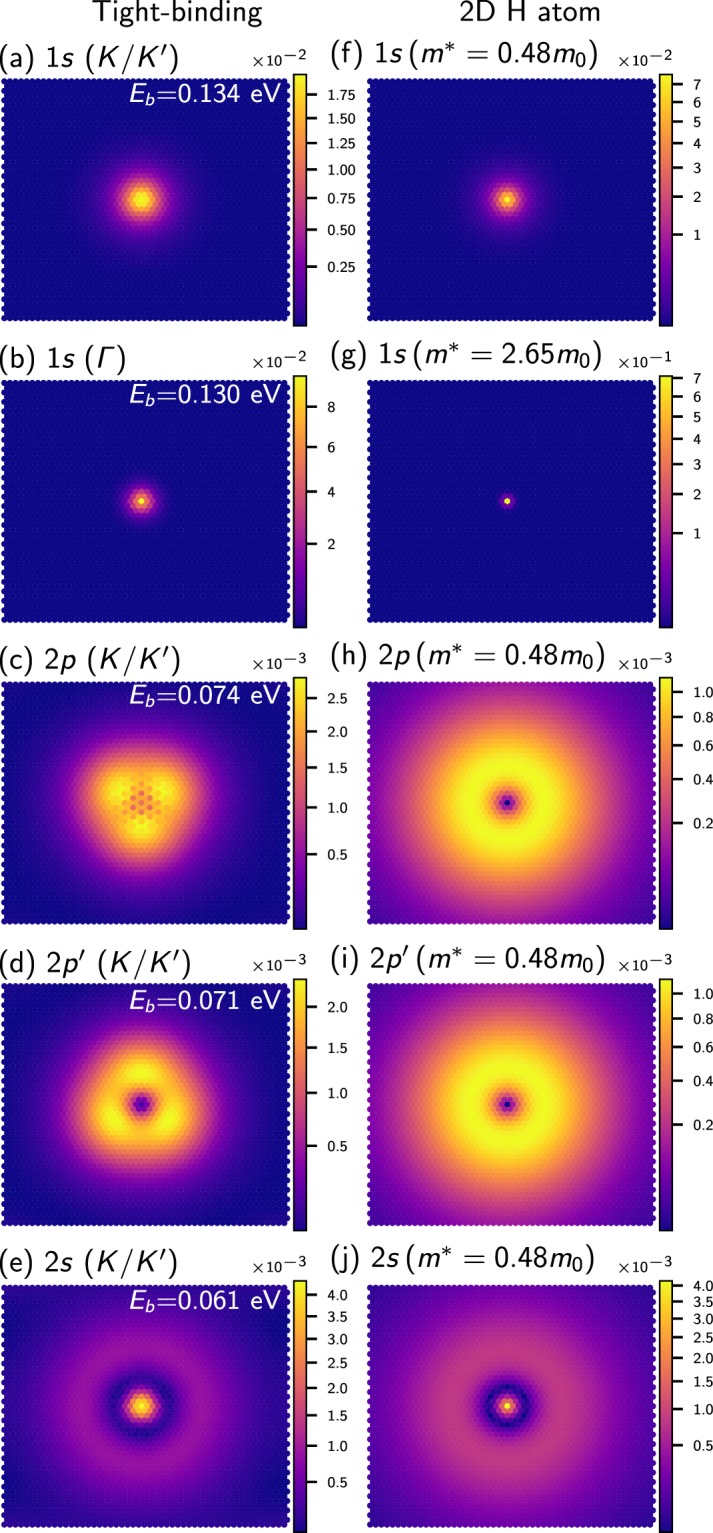
(**a**–**e**) Squared wavefunctions of bound impurity states (TB model with RPA-screened potential), for an impurity charge *Q* = −0.3*e* placed 2 Å above the Mo site. States are labelled by their 2D hydrogenic character and origin in the BZ, found by projection onto the unperturbed states (see Supplementary Material). The corresponding binding energies *E*_b_ with respect to the VBM are given in white. (**f**–**j**) 2D hydrogenic states with a nuclear charge of *Q* = −0.3*ζ e* (with *ζ* being the ratio of the screened and unscreened potentials at *r* = 0 in Fig. 1) for comparison, labelled by the effective mass of the VBM from which the corresponding states in (**a**–**e**) originate.

Figure 2(a–e) show the wavefunctions (specifically, their squared magnitudes sampled at the Γ-point of the first Brillouin zone) of the five most strongly bound impurity states for an adatom with *Z* = −0.3, situated *d* = 2 Å above the Mo-site, as calculated from our tight-binding model with an RPA-screened impurity potential. To label the impurity states, we compare them to the 2D hydrogenic states^37^. While the two most strongly bound impurity states (Fig. 2(a,b)) have 1*s* character, the states in Fig. 2(c,e and d) resemble the 2*p* and 2*s* states of the 2D hydrogen atom, respectively. We also present the corresponding 2D hydrogenic states in Fig. 2(f–j) for a nuclear charge *Q* = −0.3*ζ*, where *ζ* ≈ 0.26 is the ratio of the screened and unscreened potentials at *r* = 0 in Fig. 1. Surprisingly, the more strongly bound 1*s* states of Fig. 2(a) is significantly *more delocalized* with an impurity radius of *a*_imp_ = 12.6 Å than the less strongly bound 1*s* state in Fig. 2(b), which has a radius of *a*_imp_ = 5.19 Å. We determine *a*_imp_ by fitting the impurity state to an exponential decay as in Eq. 7, and extracting the inverse decay scale $\alpha ={a}_{{\rm{imp}}}^{-1}$. The 2*p* impurity states exhibit an angular modulation caused by the trigonal warping of the valence states near the band edge^41^. Note that the modulation is different for the two 2*p* states and we therefore label the second state distinctly as 2*p*′. In contrast to the 2D hydrogen atom, the 2*s*, 2*p* and 2*p*′ are not degenerate, as indicated by their binding energies given in the top right corner of Fig. 2(a–e), because the impurity potential is screened and no longer follows a simple 1/*r* behaviour.

**Figure 3:**
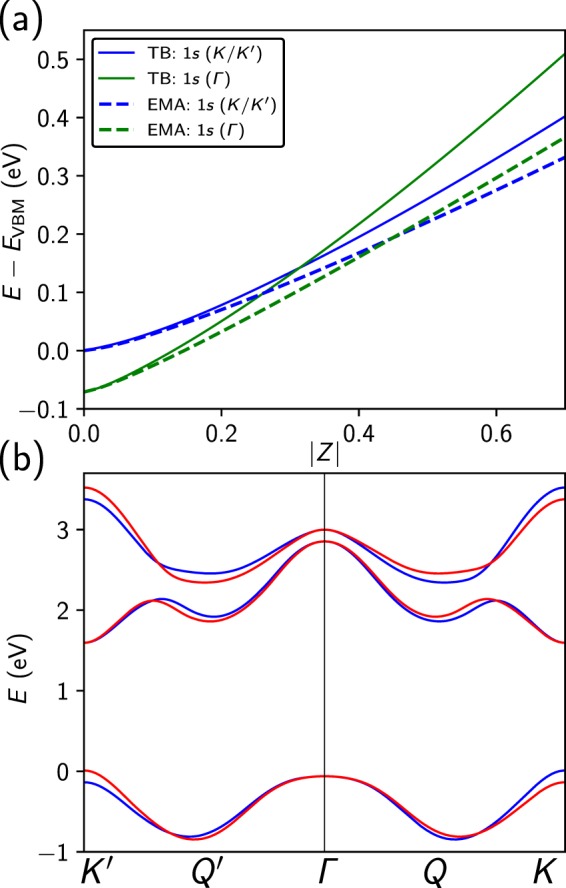
(**a**) Binding energy *E*_b_ = *E* − *E*_VBM_ of the 1*s* (*K*/*K*′) (blue) and 1*s* (Γ) (green) impurity states as a function of adatom charge *Z* for negatively charged adatoms on MoS_2_ from tight-binding calculations (solid lines) and the effective mass approximation (EMA) (dashed lines). (**b**) Tight-binding band structure, where bands with spin-up (spin-down) character are in red (blue).

To further analyze the impurity states, we projected their wavefunctions onto unperturbed states of the MoS_2_ monolayer (see Supplementary Materials for details) and find that the most strongly bound 1*s* state and also the 2*p* and 2*s* states are composed of valence states from the *K* and *K*′ points of the MoS_2_ bandstructure, see Fig. 3(b). In contrast, the second 1*s* state originates from the valence band near the Γ-point of the unperturbed band structure. We label the states in Fig. 2(a–e) by their origin in the Brillouin zone (BZ), in addition to their 2D hydrogenic orbital character. We have subsequently labelled Fig. 2(f–j) by the effective mass of the valence band maxima (VBM) from which the corresponding TB states originate.

Figure 3(a) shows the dependence of the impurity state binding energies *E*_b_ = *E* − *E*_VBM_ (energy *E* with reference to the primary valence band maximum *E*_VBM_) on the adatom charge *Z* for negatively charged adatoms. We have fitted the 1*s* binding energies to a power law of the form −*B* + *AZ*^*η*^, see Table 1, where *B* = 0 for 1*s* (*K*/*K*′) and *B* = 0.071 eV for 1*s* (Γ), and find that the 1*s* (*K*/*K*′) and 1*s* (Γ) states have exponents of *η* = 1.30 and *η* = 1.25, respectively. These are significantly smaller than the exponent for a 2D hydrogen atom where the binding energy is given by $E(Z)=-\,4\frac{{m}^{\ast }}{{m}_{0}}{Z}^{2}$ Ry. Interestingly, the different Z-dependences of the 1*s* (*K*/*K*′) and 1*s* (Γ) binding energies result in a *crossover* at *Z* = −0.32, where the order of the two states switches. As the character of 1*s* (*K*/*K*′) is dominated by Mo 4*d*_*xy*_ and $4{d}_{{x}^{2}-{y}^{2}}$ orbitals, while Mo $4{d}_{Z}2$ orbitals make up the 1*s* (Γ) state^1^, our calculations suggest the possibility of controlling the orbital character of low-lying electronic states via defect engineering with potentially interesting consequences for optical properties.

**Table 1:** Coefficients of acceptor state binding energy fits given by *E*_b_ = −*B* + *AZ*^*η*^ from tight-binding (TB) and effective mass theory (EMA) with the Keldysh model.

	*A* (eV)	*η*	*a*_imp_(−0.3) (Å)
TB: 1*s* (*K*/*K*′)	0.641	1.30	12.6
EMA: 1*s* (*K*/*K*′)	0.519	1.24	15.9
TB: 1*s* (Γ)	0.907	1.25	5.19
EMA: 1*s* (Γ)	0.661	1.15	6.65

All energies are referenced to the valence band maximum. We also show the impurity state radius *a*_imp_(*Z*) = *α*^−1^(*Z*) of the 1*s* states for *Z* = −0.3.

To further analyze the results of the tight-binding calculations, the bound impurity states were studied with effective mass theory. Specifically, we determined the impurity states associated with the subzones near Γ, *K* and *K*′ using Eq. (7). For the acceptor states, each subzone acts as an independent 2D hydrogen-like system as the different spin states of the degenerate valence band maxima at *K* and *K*′ prohibit interactions between the subzones. The resulting binding energies agree reasonably well with the tight-binding results, see dashed lines in Fig. 3(a) and Table 1. We see that the discrepancy between these two models increases with *Z*, as the RPA-screened potential in Fig. 1 is deeper than the screened potential in the Keldysh model, resulting in more strongly bound states. In particular, effective mass theory also predicts a crossover of 1*s* (*K*/*K*′) and 1*s* (Γ) near *Z* = −0.45. The binding energy of 1*s* (Γ) increases more quickly with *Z* because the effective mass near Γ is about 5.5 times larger than the effective mass near *K* or *K*′. This also explains the differences in impurity radii, see Fig. 2(a,b).

### Donor States

**Figure 4:**
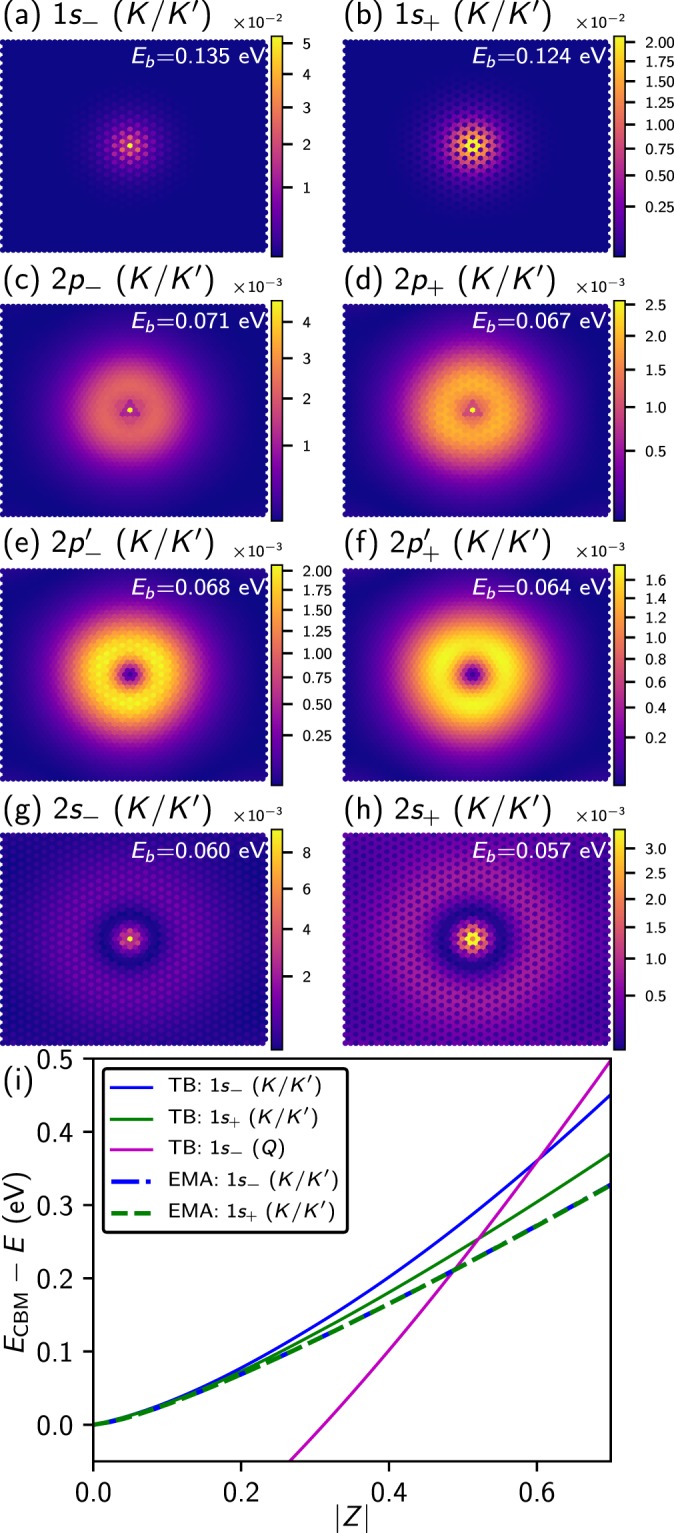
(**a**–**h**) Squared wavefunctions of bound impurity states for an impurity charge *Q* = +0.3*e* placed 2 Å above the Mo site, with binding energies *E*_b_ = *E*_CBM_ − *E* indicated (white). Hybridised states are separately labelled with ± subscripts. (i) Binding energy *E*_b_ of hybridized 1*s* (*K*/*K*′) (green and blue) and 1*s* (*Q*) (magenta) impurity states as a function of adatom charge *Z* for positively charged adatoms on MoS_2_ from TB (solid lines) and EMA (dashed lines).

Next, we study the shallow impurity states induced by positively charged adatoms. Figure 4(a–h) show the wavefunctions of the eight most strongly bound impurity states for an adatom with *Z* = 0.3 and *d* = 2 Å. The states are labelled based on their similarity to the eigenstates of the 2D hydrogen atom. In contrast to the acceptor case, we find *a pair of states* corresponding to each solution of the 2D hydrogen atom, with different binding energies, indicated at the top right corner of each subfigure in white. The states of each pair are distinguished by a “+” or “−” subscript.

Figure 4(i) shows the binding energies *E*_b_ = *E*_CBM_ − *E* of the most strongly bound states (with energy *E*) with respect to the conduction band minimum (with energy *E*_CBM_) as function of the impurity charge *Z*. At low values of *Z*, the 1*s*_−_(*K*/*K*′) and 1*s*_+_(*K*/*K*′) states are almost degenerate, but their binding energy difference increases with increasing *Z*. A third impurity state originating from the local conduction band minimum at the 6 *Q* points of the Brillouin zone crosses the two 1*s* (*K*/*K*′) states near *Z* = 0.6 and becomes the most strongly bound state for higher values of *Z*. The crossover is again caused by the larger effective mass at *Q* point compared to the *K* and *K*′ points. We have fitted the binding energies of these states to a power law of the form *B* + *AZ*^*η*^, see Table 2, where *B* = 0 for states from *K*/*K*′ and *B* = 0.267 eV for states from the *Q*-points. As for the acceptor impurity states, the exponents of the donor states are significantly smaller than the 2D hydrogen value *η* = 2.

**Table 2:** Coefficients of donor state binding energy fits given by *E*_b_ = −*B* + *AZ*^*η*^ from tight-binding (TB) and effective mass theory (EMA) with the Keldysh model.

	*A* (eV)	*η*	*a*_imp_(0.3) (Å)
TB: 1*s*_−_(*K*)	0.743	1.42	12.7
EMA: 1*s*_−_(*K*)	0.513	1.24	15.4
TB: 1*s*_+_(*K*)	0.588	1.29	16.4
EMA: 1*s*_+_(*K*)	0.511	1.24	15.4
TB: 1*s*_−_(*Q*)	1.217	1.30	—

All energies are referenced to the valence band maximum. We also show the impurity state radius *a*_imp_(*Z*) = *α*^−1^(*Z*) for *Z* = 0.3.

Again, we compare the tight-binding results to effective mass theory. We first determine the subzone envelope functions, Eq. (7), for the regions near the critical points at *K* and *K*′. In contrast to the valence bands, there is no spin-orbit splitting of the conduction band states at *K* and *K*′. As a consequence, the conduction band states at *K* and *K*′ with equal spin are degenerate and this gives rise to the observed pairs of impurity states with same symmetry in Fig. 4. The subzone impurity states can couple and the resulting binding energy splitting is given by^36^

8 $${{\rm{\Delta }}}_{KK^{\prime} }\approx 2|{\varphi }_{1s,{\bf{K}}}^{\ast }(r=0){\varphi }_{1s,{\bf{K}}^{\prime} }(r=0)V({\bf{q}}={\bf{K}}-{\bf{K}}^{\prime} )|.$$

We evaluate the splitting with the Keldysh approximation for *V*, using the Fourier transform of the screened Coulomb potential in the Keldysh model. We find that the splitting is several orders of magnitude smaller than the splitting found in the tight-binding model. This discrepancy is caused by the inaccurate behaviour of the Keldysh model at large wave vectors, which is shown in the inset of Fig. 1, where the vertical black line indicates |**K** − **K**′|. We note, however, that for such large wavevectors (corresponding to positions in the immediate vicinity of the impurity) the use of the 2D dielectric function can cause inaccuracies, as Eq. 2 assumes that the distance from the impurity is significantly larger than the width of the MoS_2_ sheet^29^. We show the binding energies, found from effective mass theory using the Keldysh screening model for the splitting (see Fig. 4(i) as blue dashed an green dot-dashed lines). The fitting parameters of the binding energies to a power law are compared to the tight-binding results in Table 2.

The 1*s* impurity state wavefunctions from effective mass theory are given by

9 $${{\rm{\Psi }}}_{1{s}_{\pm }(K/K^{\prime} )}({\bf{r}})=\frac{1}{\sqrt{2}}({\varphi }_{1s,{\bf{K}}}(r){\psi }_{{\bf{K}}}({\bf{r}})\pm {\varphi }_{1s,{\bf{K}}^{\prime} }(r){\psi }_{{\bf{K}}}({\bf{r}})),$$

where ${\psi }_{K/K^{\prime} }({\bf{r}})$ denote the Bloch states of the unperturbed MoS_2_ band structure at *K* and *K*′. Notably, the states with an *s*-character (Fig. 4(a,b,d and h)) exhibit an intensity modulation with a period of three unit cells along the directions connecting nearest neighbours. Projecting the impurity states onto unperturbed Bloch states reveals that all states originate from both the *K* and *K*′ points of the Brillouin zone, where the minimum of the conduction band occurs, see Fig. 3(b). The corresponding probability densities contain a term with a cos((**K** − **K**′) ·**r**) factor which gives rise to the oscillatory pattern in Fig. 4(a,e,d,h). In contrast to the impurity states with *s*-character which derive from unperturbed states directly at *K* and *K*′, the states with *p*-character mostly derive from conduction band states in the vicinity of the band edges. As a consequence, the coupling between *K* and *K*′ is weaker for the *p*-states and the spatial modulation is not observed. We find that this modulation does not occur when the defect is not placed on the transition-metal site.

### Local density of states

Scanning tunnelling spectroscopy (STS) provides spatially-resolved information about the electronic structure of surfaces and has been used to study the properties of shallow impurity states induced by charged adatoms experimentally. The d*I*/d*V* curves obtained in STS are often assumed to be proportional to the local density of states (LDOS) of the sample. We have calculated the LDOS for values of *Z* and *d* that represent lithium (Li) and carbon (C) atoms adsorbed on a MoS_2_. For Li, Chang *et al*. found an impurity charge of *Z*_Li_ = 0.63 from a Bader charge analysis^42^ of the DFT charge density^18^. Using a similar procedure, Ataca *et al*. determined *Z*_C_ = −0.58 for a C atom adsorbed to MoS_2_ above the Mo site^19,43^. We modelled adsorbed atoms sitting above the Mo site at a height of *d*_Li_ = 3.1 Å and *d*_C_ = 1.58 Å^17–19,43^. Screening by a SiO_2_ substrate is included via a substrate dielectric function of 3.7.

**Figure 5:**
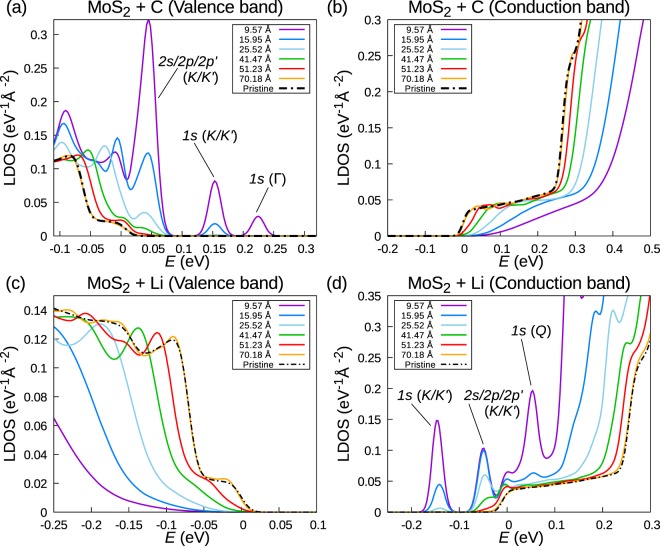
(**a**,**b**) LDOS of a lithium (Li) adatom on MoS_2_ (+SiO_2_ substrate) near the (**a**) valence band and (**b**) conduction band edge. (**c**,**d**) LDOS of a carbon (C) adatom on MoS_2_ (+SiO_2_ substrate) near the (**c**) valence band and (**d**) conduction band edge. Results are shown for several distances from the impurity. In each graph, the zero of energy is set to the band edge of the unperturbed MoS_2_.

Figure 5(a,b) show the tight-binding LDOS for a C adatom on MoS_2_ in the vicinity of valence band maximum and the conduction band minimum, respectively. A 6 × 6 *k*-point mesh and a Gaussian broadening of 0.01 eV were used. Near the VBM, several peaks originating from bound acceptor states can be observed in the band gap. The peak from 1*s* (Γ) disappears more quickly as a function of distance from the adatom than the 1*s* (*K*/*K*′) peak. This is a consequence of the stronger localization of this state, see Fig. 2. At a distance of ~66 Å from the adatom, the LDOS of the perturbed system has converged to the LDOS of the pristine TMD. In the vicinity of the CBM, no impurity states are present. However, the screened potential created by the adatom leads to a shift of the unperturbed LDOS.

Figure 5(c,d) show the tight-binding LDOS for a Li adatom on MoS_2_ in the vicinity of valence band maximum and the conduction band minimum, respectively. The peaks near the CBM in the vicinity of the adatom originate from bound donor states and can be observed up to a distance of ~25 Å from the adatom. Note that the splitting of the two impurity states from the *K* and *K*′ points is too small to be resolved. No impurity state peaks are found in the vicinity of the VBM, but again the impurity potential causes a shift of the TMD LDOS.

## Conclusions

In summary, we have studied the electronic properties of charged defects in transition-metal dichalcogenides. Using tight-binding simulations with screened impurity potentials on unit cells containing up to 8,000 atoms, we have calculated the binding energies and wave functions of shallow impurity bound states. Our key finding is that the orbital character of the lowest lying impurity states depends sensitively on the magnitude of the defect charge. For acceptor states, i.e., negatively charged defects, a crossover of impurity states with different orbital characters occurs at a critical defect charge of *Q* = −0.32 *e* (with *e* being the proton charge). For defect charges above this value, the lowest impurity state from the Γ valley of the TMD band structure, which is dominated by contributions from Mo $4{{\rm{d}}}_{{{\rm{z}}}^{2}}$ orbitals, is more strongly bound than the degenerate impurity states from the *K* and *K*′ valleys which are dominated by Mo 4d_*xy*_ and Mo $4{{\rm{d}}}_{{x}^{2}-{y}^{2}}$ orbitals. For donor states, i.e., positively charged defects, a crossover between hybridized impurity states from the *K* and *K*′ valleys and impurity states from the *Q* valleys occurs at a critical impurity charge of +0.6 *e*. To understand the competition between different impurity states, we analyze their properties using effective mass theory. We find that the impurity binding energies can be described by power laws of the defect charge, but with significant deviations from hydrogenic behaviour due to screening. Importantly, the prefactor of the power law is determined by the effective mass and the significant differences of the effective masses in the different valleys of the TMD band structure give rise to the observed crossovers. Our calculations thus establish the defect charge as an important control parameter for tuning the electronic structure of TMDs via defect engineering.

## Electronic supplementary material

Supplementary information

## Data Availability

The datasets generated during and/or analysed during the current study are available from the corresponding author on reasonable request.
