# Supplementary material for: Tuning electronic properties of transition-metal dichalcogenides via defect charge
Source: Sci Rep. 2018 Sep 11;8:13611. doi: 10.1038/s41598-018-31941-1 (PMC6134151; doi:10.1038/s41598-018-31941-1)
Supplement: Supplementary file 1 — Supplementary information [file 41598_2018_31941_MOESM1_ESM.pdf]

Supplementary Material: Tuning electronic properties of transition-metal dichalcogenides via defect charge

Martik Aghajanian, Arash A. Mostofi, and Johannes Lischner*

Departments of Physics and Materials, and the Thomas Young Centre for Theory and Simulation of Materials, Imperial College London, London, SW7 2AZ

E-mail: j.lischner@imperial.ac.uk

Phone: +44 (0)20 7594 9949

Ab initio Adatom Potential

The screened potential of a charged adatom on a molybdenum disulfide (MoS_2) monolayer is obtained by first calculating the dielectric matrix $\varepsilon_{\mathbf{G}\mathbf{G}'}(\mathbf{q})$ of an infinite stack of MoS_2 sheets and then calculating $\varepsilon_{2\mathrm{D}}^{-1}(\mathbf{q})$ using[1]

$$\varepsilon_{2\mathrm{D}}^{-1}(\mathbf{q}) = \frac{q}{2\pi e^2 L_z} \sum_{\mathbf{G}_z \mathbf{G}'_z} \varepsilon^{-1}_{\mathbf{G}_z \mathbf{G}'_z}(\mathbf{q}) v_{\mathrm{trunc}}(|\mathbf{q} + \mathbf{G}'_z|), \tag{S1}$$

where $\mathbf{G}_z$ and $\mathbf{G}'_z$ denote reciprocal lattice vectors along the out-of-plane (z) direction, v_{trunc} is a slab-truncated Coulomb interaction[2] and L_z denotes the distance between the stacked sheets. To do this, we first perform density-functional theory (DFT) calculations within the generalized gradient approximation (GGA), using the Perdew-Burke-Ernzerhof (PBE) exchange-correlation functional and optimized norm-conserving Vanderbilt pseudopotentials.[3] Calculations were carried out using the Quantum Espresso software package.[4] To

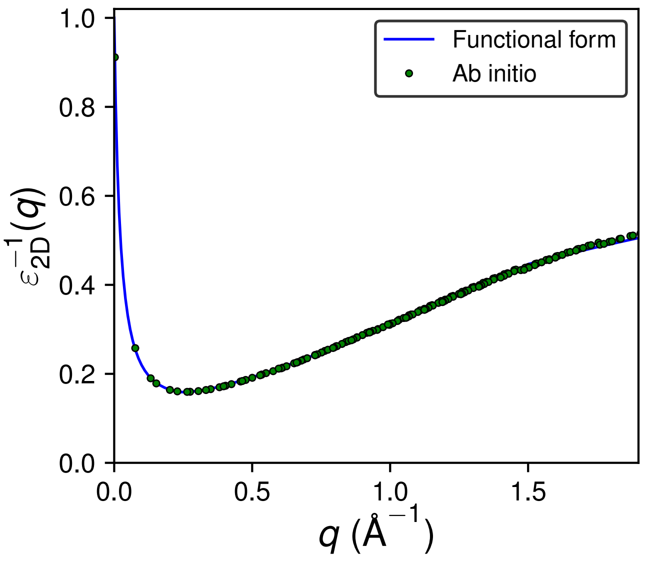

Figure S1: Inverse dielectric function of MoS_2 for both a fitted functional form (blue curve) and sampled ab initio (green markers).

determine the ground state electron density, we use a 14×14 Γ-centred k-point mesh and an 80 Ry plane-wave cutoff. The stacked MoS_2 sheets are separated by $L_z = 15.95$ Å in the out-of-plane direction. Next, the inverse dielectric matrix $\varepsilon^{-1}_{\mathbf{GG'}}(\mathbf{q})$ is calculated using the BerkeleyGW software package,[5] on a 30×30 q-point mesh using a plane-wave cut-off of 30 Ry and we sum over 2587 unoccupied states. The sampled points are shown in Fig. S1 as green circles, showing that the inverse dielectric function is isotropic at small wavevectors. Having determined $\varepsilon^{-1}_{\mathrm{2D}}(\mathbf{q}) = \varepsilon^{-1}_{\mathrm{2D}}(q)$ from first principles on a discrete q-point mesh, we fit the high-q and low-q regions to a functional form, and express the remaining mid-q region as the sum of the inverse dielectric function of the tight-binding calculation and a correction in order to perform integrations in reciprocal space more easily. For $q < q_{\mathrm{low}}$ with $q_{\mathrm{low}} \approx 0.23$ Å^{-1}, the sampled dielectric function is fitted to $\tanh(x)$, as this has been previously used to describe the long-range screening of thin-film semiconductors.[6] This takes the form:

$$\varepsilon_{\mathrm{low}}(q) = \kappa_1 \tanh\left(\frac{qh_1}{2} + \frac{1}{2}\ln\left|\frac{\kappa_1+1}{\kappa_1-1}\right|\right), \tag{S2}$$

where we find $\kappa_1 = 6.68$ and $h_1 = 13.17$ Å. For $q_{\text{low}} \leq q < q_{\text{high}}$, where $q_{\text{high}} = 1.5\ \text{Å}^{-1}$, we represent $\varepsilon^{-1}_{\text{2D}}(\mathbf{q})$ using the dielectric function of the three-band tight-binding model, given by

$$\varepsilon_{\text{TB}}(\mathbf{q}) = 1 - \frac{v_{\mathbf{q}}}{\Omega} \sum_{nn'} \sum_{\mathbf{k}\in\text{BZ}} \frac{(f_{n\mathbf{k}} - f_{n'\mathbf{k}+\mathbf{q}})|M_{nn'}(\mathbf{k},\mathbf{q})|^2}{E_{n\mathbf{k}} - E_{n'\mathbf{k}+\mathbf{q}}}, \tag{S3}$$

where $v_{\mathbf{q}} = 2\pi/q$ is 2D Fourier transform of the Coulomb potential, Ω is the unit cell area, and $M_{nn'}(\mathbf{k},\mathbf{q}) = \langle\psi_{n\mathbf{k}}|e^{-i\mathbf{q}\cdot\mathbf{r}}|\psi_{n'\mathbf{k}+\mathbf{q}}\rangle$ is the matrix element. For $q < 1.5\ \text{Å}^{-1}$, we find that $\varepsilon^{-1}_{\text{TB}}(\mathbf{q}) \equiv 1/\varepsilon_{\text{TB}}(\mathbf{q})$ is highly isotropic and we carry out an angular average to obtain $\bar{\varepsilon}^{-1}_{\text{TB}}(q)$. To include the effect of the other bands on screening, we employ the correction proposed by Wehling *at al.*[7] which captures to electrostatic screening of a thin film with thickness h_2 and dielectric constant κ_2 at long wavelengths:

$$\delta\varepsilon^{-1}(q) = \frac{1}{\kappa_2} \frac{\kappa_2 + 1 + (\kappa_2 - 1)\, e^{-qh_2}}{\kappa_2 + 1 - (\kappa_2 - 1)\, e^{-qh_2}} - 1. \tag{S4}$$

The parameters $\kappa_2 = 0.69$ and $h_2 = 0.73$ Å were fitted to the ab initio inverse dielectric function. At large values of $q \geq q_{\text{high}}$, the tail of the dielectric function is fitted to $\varepsilon_{\text{high}}(q) = 1 + \chi_c/q$, where $\chi_c = 1.83\ \text{Å}^{-1}$. In summary, we fit $\varepsilon^{-1}_{\text{2D}}(q)$ in Eq. S1 to the functional form:

$$\varepsilon^{-1}_{\text{2D}}(q) = \begin{cases} \varepsilon^{-1}_{\text{low}}(q), & q < q_{\text{low}}, \\ \bar{\varepsilon}^{-1}_{\text{TB}}(q) + \delta\varepsilon^{-1}(q; h, \kappa), & q_{\text{low}} \leq q < q_{\text{high}}, \\ \varepsilon^{-1}_{\text{high}}(q), & q \geq q_{\text{high}}, \end{cases}$$

ensuring continuity between intervals. The corrected dielectric function $\varepsilon^{-1}_{\text{2D}}(q)$ is shown in Fig. S1, comparing ab initio results with the corrected dielectric function.

Reciprocal-space Impurity Envelopes

To identify the origin in the Brillouin zone (BZ) of impurity states, we construct unperturbed $N \times N$ supercell eigenstates using unit cell states $|\Psi^{\mathrm{UC}}_{\mathbf{g}m}\rangle$ which fold onto the Γ point, such that $\mathbf{g} = \mathbf{G}/N$ lies in the first BZ of the unit cell system and m is the band index. For each

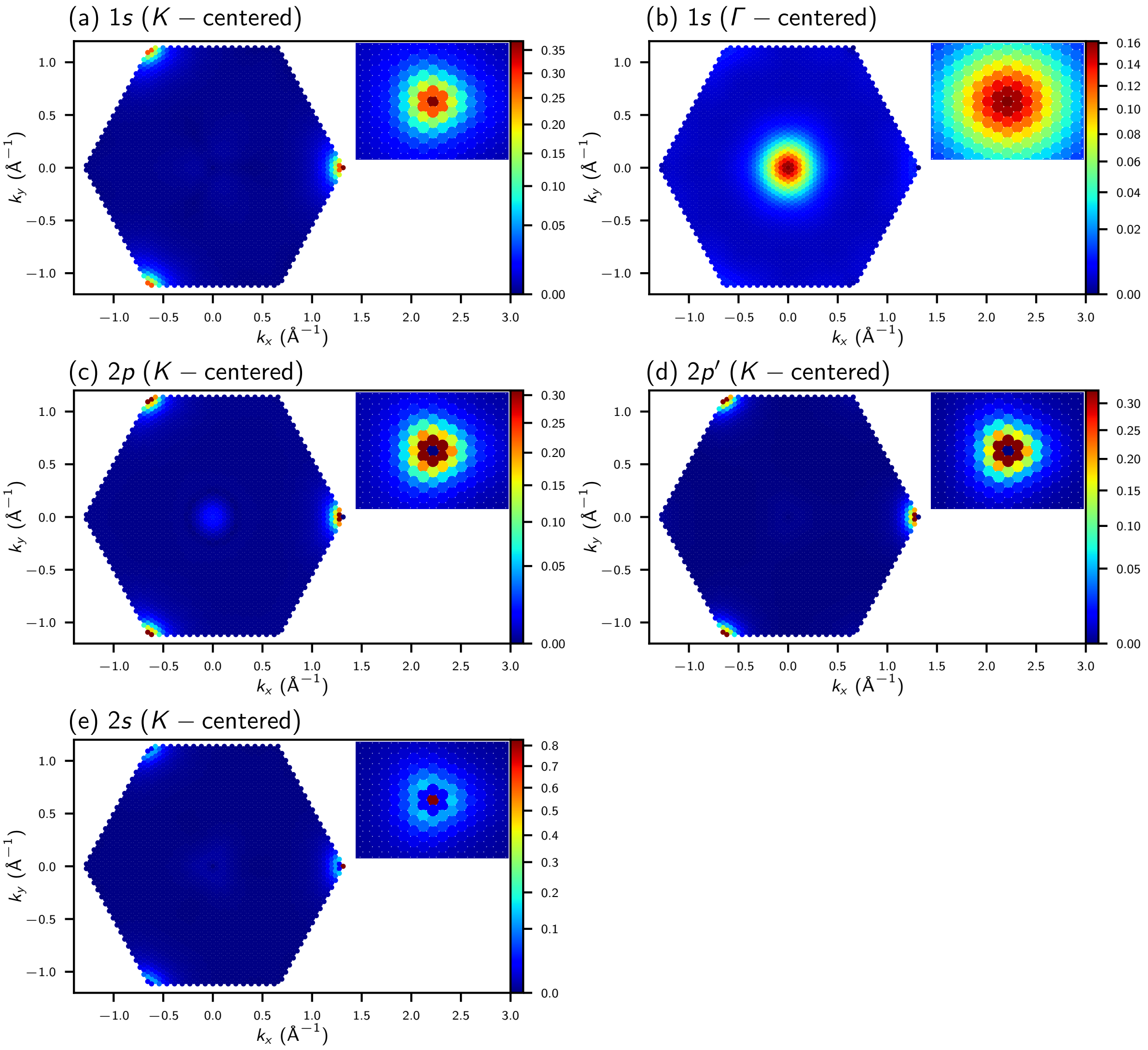

Figure S2: Projections of top five impurity states in the Brillouin Zone, for a $Z = -0.3$ acceptor charge placed $d = 2$ Å above a MoS_2 monolayer. The inset shows the projections centred on their respective high-symmetry points.

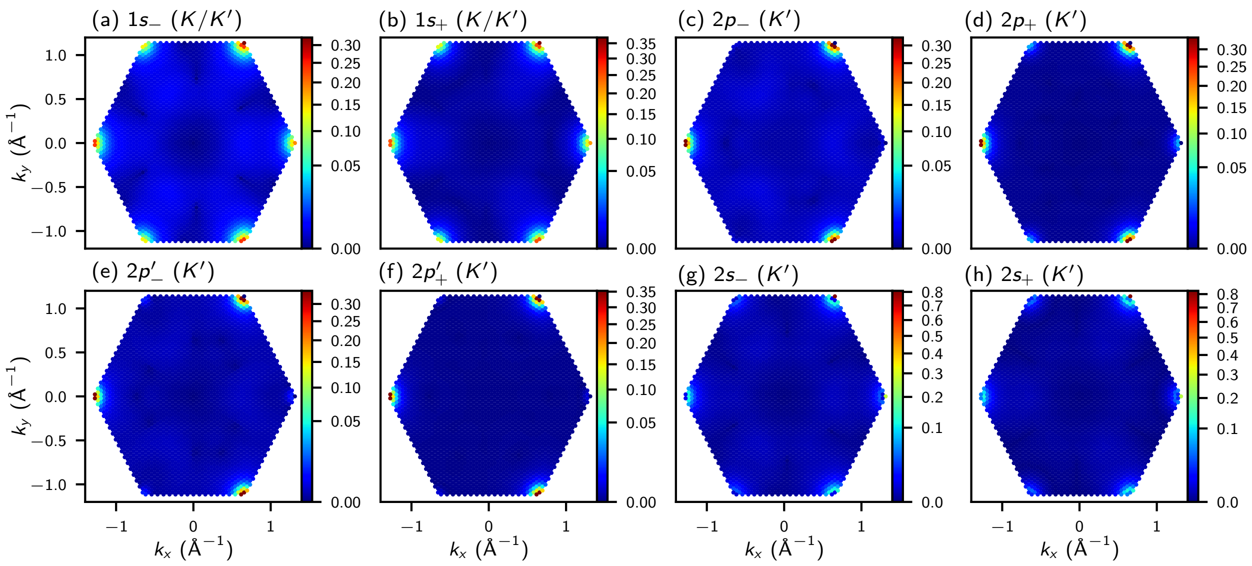

Figure S3: Projections of top eight impurity states in the Brillouin Zone, for a $Z = 0.3$ donor charge placed $d = 2$ Å above a MoS_2 monolayer.

$\mathbf{g}$, we create the set of eigenstates

$$|\Psi^{(N)}_{\Gamma_n}\rangle \equiv |\Psi^{(N)}_{\mathbf{g}m}\rangle = \frac{1}{N}\begin{pmatrix} e^{i\mathbf{g}\cdot\tau_0} \\ e^{i\mathbf{g}\cdot\tau_1} \\ \vdots \\ e^{i\mathbf{g}\cdot\tau_{N-1}} \end{pmatrix} \bigotimes |\Psi^{\mathrm{UC}}_{\mathbf{g}m}\rangle,$$

where τ_i is position of the ith unit cell in the supercell, and n now orders the folded eigenstates at Γ in energy. We project the eigenstates of the perturbed supercell onto the set $\left\{|\Psi^{(N)}_{\mathbf{g}m}\rangle\right\}$ to determine the origin $\mathbf{g}$ in the BZ of the impurity states. For acceptor states at $Z = -0.3$, in Fig. S2 we show the projections in the BZ. These are centered on their respective origins in the BZ, and demonstrate interesting localisation. For the $1s$ states, we see that the Γ state is more delocalized in $k-$space than the state from K. The $2s$ states, originating from K, demonstrate three-fold anisotropy attributed to the trigonal warping of the valence bands at K. This clear anisotropy manifests itself in the three-fold symmetric acceptor impurity state wavefunctions in the main text, with orientation determined by the correspondence between the crystal lattice vectors and reciprocal lattice vectors. While Fig. S2 shows only

the absolute square of the projection for the $2p$ and $2p'$, the phases for the $2p$ and $2p'$ are opposite in sign and similar in value, resulting in a π rotation of the $2p$ state onto the $2p'$ state. Anisotropy in the reciprocal-space impurity envelope occurs most prominently at inverse scales ≈ 0.06 Å^{-1} – 0.16 Å^{-1}, corresponding to trigonal lobes at ≈ 20 Å from the impurity bonding site. This anisotropy is not as prominent in the $2s$ state and negligible in the $1s$ state, which results in more isotropically distributed impurity wavefunctions. We have not shown the states at K', as they contain the same information as Fig. S3. When the K and K' fold onto Γ, states from both points have a set of $1s, 2s, 2p/p'$ states with opposite spin in the three-band model. We also show the projection of the donor states for a charge of $Z = 0.3$.

References

(1) Qiu, D. Y.; da Jornada F. H.,; Louie, S. G. *Phys. Rev. B* **2016**, *93*.

(2) Ismail-Beigi, S. *Phys. Rev. B* **2006**, *73*.

(3) Hamann, D. R. *Phys. Rev. B* **2013**, *88*, 1–10.

(4) Giannozzi, P. et al. *J. Phys.: Cond. Matt.* **2009**, *21*.

(5) Deslippe, J.; Samsonidze, G.; Strubbe, D. A.; Jain, M.; Cohen, M. L.; Louie, S. G. *Comp. Phys Comms.* **2012**, *183*, 1269 – 1289.

(6) Keldysh, L. V. *JETP Lett.* **1979**, *29*, 658.

(7) Wehling, T. O.; aolu, E.; Friedrich, C.; Lichtenstein, A. I.; Katsnelson, M. I.; Blgel, S. *Phys. Rev. Lett.* **2011**, *106*.
